# Performance of the QuickVue Influenza A+B Rapid Test for Pandemic H1N1 (2009) Virus Infection in Adults

**DOI:** 10.1371/journal.pone.0028089

**Published:** 2011-12-01

**Authors:** Wolfgang Poeppl, Harald Herkner, Heinz Burgmann, Tom Pustelnik, Gerhard Mooseder, Theresia Popow-Kraupp, Monika Redlberger-Fritz

**Affiliations:** 1 Department of Infectious Diseases and Tropical Medicine, Medical University of Vienna, Vienna, Austria; 2 Department of Emergency Medicine, Medical University of Vienna, Vienna, Austria; 3 Department of Dermatology and Tropical Medicine, Military Hospital Vienna, Vienna, Austria; 4 Department of Virology, Medical University of Vienna, Vienna, Austria; University of Hong Kong, Hong Kong

## Abstract

To investigate the diagnostic accuracy of the QuickVue® Influenza A+B rapid test we conducted a prospective observational study in which this rapid test was compared with a real-time reverse transcription polymerase chain reaction (RT-PCR) for pandemic influenza A H1N1 (2009) infection in Austrian adults. The sensitivity, specificity, and positive and negative predictive values of the QuickVue test compared with the RT-PCR were 26% (95% CI 18–35), 98% (95% CI 92–100), 94% (95% CI 80–99) and 50% (95% CI 42–58), respectively. The prevalence of pandemic H1N1 (2009) virus infection among the 209 patients included in the study was 57%. Our data suggest that a positive QuickVue test provides considerable information for the diagnosis of pandemic influenza A H1N1 (2009) virus infection in young adults but that a negative QuickVue test result should, if relevant for patient management or public health measures, be verified using PCR.

## Introduction

Influenza is a seasonal viral infection associated with significant morbidity and mortality during local outbreaks and epidemics [1]. Early diagnosis is essential for application of preventive strategies and initiation of antiviral therapy in patients at risk of complications [2]. Real-time reverse transcription (RT) PCR is the current method of choice for detection of influenza virus infection, with a reported sensitivity of 98–100% and a specificity of 100% [3]. However, RT-PCR is expensive and is rarely available in the local primary care setting.

For timely diagnosis, physicians rely on point-of-care testing that is easy to perform and yields results within minutes, thus commercially available rapid influenza diagnostic tests (RIDTs) are widely used in the primary care setting. Nevertheless, RIDTs do not distinguish among influenza A virus subtypes and test sensitivity might therefore vary [4]. The recent appearance and spread of novel influenza H1N1 virus has highlighted the need to evaluate commercially available and widely used RIDTs for their ability to detect these viral antigens in clinical respiratory specimens [4].

There have been several studies on the validity of RIDTs for diagnosis of pandemic influenza A H1N1 (2009) virus. Results have revealed wide variability, with sensitivities ranging from 10% to 75% [2], [4], [5], [6], [7], [8], [9], [10], [11], [12]. As with all screening tests, the positive and negative predictive values (PPVs and NPVs) of RIDTs depend on the prevalence of the disease in the population being tested. Further, differences among age groups, differing study designs and sample sizes, as well as heterogeneous sample collection, cause difficulties in comparison of such studies [4].

We conducted a prospective observational study to investigate the performance of the QuickVue® Influenza A+B rapid test (Quidel Corp., San Diego, CA, USA) in comparison with a real-time RT-PCR for detection of pandemic influenza A H1N1 (2009) virus infection in adults during the 2009 H1N1 pandemic in Austria.

## Materials and Methods

### Ethics statement

The study protocol was approved by the Ethics Committee of the Medical University Vienna and the local institutional review board of the Military Hospital Vienna. Because all samples were collected as per standard of care for routine diagnostic testing and all data were analyzed anonymously, the requirement for informed consent was waived by the institutional review board.

### Patients

The study was conducted at the Military Hospital Vienna during the 2009 influenza pandemic. All patients with clinical suspicion of pandemic influenza A H1N1 (2009) virus infection were routinely tested using the QuickVue Influenza A+B rapid test and RT-PCR. A total of 208 male and one female patient were included between 20 September 2009 and 26 January 2010. The mean age of patients with influenza-like illness was 20 years (range 17–38 years).

### Clinical samples and viral detection methods

Sample collection and testing with the QuickVue kit was performed by physicians previously instructed by the hospital's hygiene team. Swabs were taken from the oropharynx and nares. The rapid test was performed with a first respiratory specimen using the foam swab included in the kit. A second sample for RT-PCR testing was collected simultaneously using a sterile Dracon swab.

The rapid tests were performed immediately on-site according to the manufacturer's instructions. Samples for RT-PCR testing were placed in 0.9% sodium chloride solution and immediately transferred to the Department of Virology, Medical University Vienna.

Viral RNA was isolated from respiratory specimens using the QIAamp Viral RNA Mini Kit (QIAGEN, Hilden, Germany). Reverse transcription was performed using an iScript cDNA synthesis kit (Bio-Rad, CA, USA) according to the manufacturer's instructions.

The real-time PCR was then performed in a LightCycler 480 (Roche Diagnostics, Mannheim, Germany) using Taq Man (R) Universal PCR Mastermix; using primer H1-1076Fw: 5′-CAG GGA TGG TAG ATG GAT GG-3′, H1-1165Rv: 5′-TGG CAT TCT GTG TGC TCT TC-3′and probe 1120P: 5′ FAM-CAG GGG TCA GGA TAT GCA GCC G-3′TAMRA (primer positions according to GenBank accession no. FJ966974).

Briefly, 1 µl viral c-DNA was added to the following reaction mixture: 12,5 µl Taq Man® Universal PCR Mastermix (Applied Biosystems, USA), 0,4 µl forward primer (25 pmol), 0,4 µl reverse primer (25 pmol), 0,2 µl probe (10 pmol) and 6,5 µl water. The cycling conditions were: initial holds at 50°C for 3 min and 95°C for 10 min followed by 45 cycles at 95°C for 15 s, 55°C for 30 s and 77°C for 31 s.

For all PCR testing, a cycle threshold (Ct, the cycle count at which amplified product yielded a detectable fluorescent signal) <42 was interpreted as positive. Ct values are indicators of the amount of virus in the specimen, with lower values indicating higher viral loads.

To exclude false positive results in the QuickVue test due to seasonal influenza A infection, samples from patients with a positive QuickVue result for influenza A but a negative RT-PCR for pandemic influenza A H1N1 (2009) were also checked by PCR analysis for influenza A viruses (covering all influenza A virus subtypes) and in addition by subtype specific PCR's for seasonal influenza A/H1N1 and A/H3N2 virus [13].

### Statistical analysis

Data are presented as mean and range or median and 25–75% interquartile range. Categorical data are presented as absolute and relative frequencies. We used logistic regression to estimate the effect of observation time (date of sample collection during the pandemic season) on influenza prevalence, including a test for linearity.

Sensitivity, specificity, predictive values and likelihood ratios were estimated according to standard definitions, together with 95% confidence intervals based on exact standard errors. We compared Ct values between rapid test positive and negative samples using a Mann–Whitney U test.

Stata 11 for Mac (StataCorp, College Station, Tx, USA) was used for all statistical analyses.

A two-sided p value<0.05 was considered statistically significant.

## Results

Of the 209 specimens tested, 119 came from patients who had positive PCR results for pandemic influenza H1N1 (2009) virus. Among these 119 patients, the QuickVue test was positive in 31 (26%) and negative in 88 (74%) (Table 1). Of 90 specimens negative in the PCR, two (2%) were positive in the rapid test and 88 (98%) were negative. The overall prevalence of pandemic H1N1 (2009) virus infection in the 209 samples was 57%. The frequency of positive results peaked in November 2009. The proportion of positive rapid test results among all persons tested, however, decreased with each month from September 2009 to January 2010 (OR 0.26, 95% CI 0.14–0.51).

**Table 1:** Performance of the QuickVue Influenza A+B rapid test in comparison with RT-PCR in the diagnosis of pandemic H1N1 (2009) virus infection.^a^

	Rapid test positive	Rapid test negative	
**PCR positive**	31	88	129
**PCR negative**	2	88	90
	33	176	209
**Prevalence of PCR positives in sample (95% CI)**			57% (50–64)
**Sensitivity (95% CI)**			26.05% (18–35)
**Specificity (95% CI)**			97.78% (92–100)
**Positive predictive value (95% CI)**			93.94% (80–99)
**Negative predictive value (95% CI)**			50.00% (42–58)
**Positive likelihood ratio (95% CI)**			12.00 (2.88–48)
**Negative likelihood ratio (95% CI)**			0.76 (0.68–0.85)

a confidence interval (CI).

### Performance of the QuickVue Influenza A+B rapid test

The overall sensitivity, specificity, PPV and NPV of the QuickVue Influenza A+B rapid test for 2009 (H1N1) influenza in comparison with RT-PCR are presented in Table 1.

The positive and negative likelihood ratios were 12 and 0.76, respectively.

### Relation between Ct value and rapid test result

**Figure 1:**
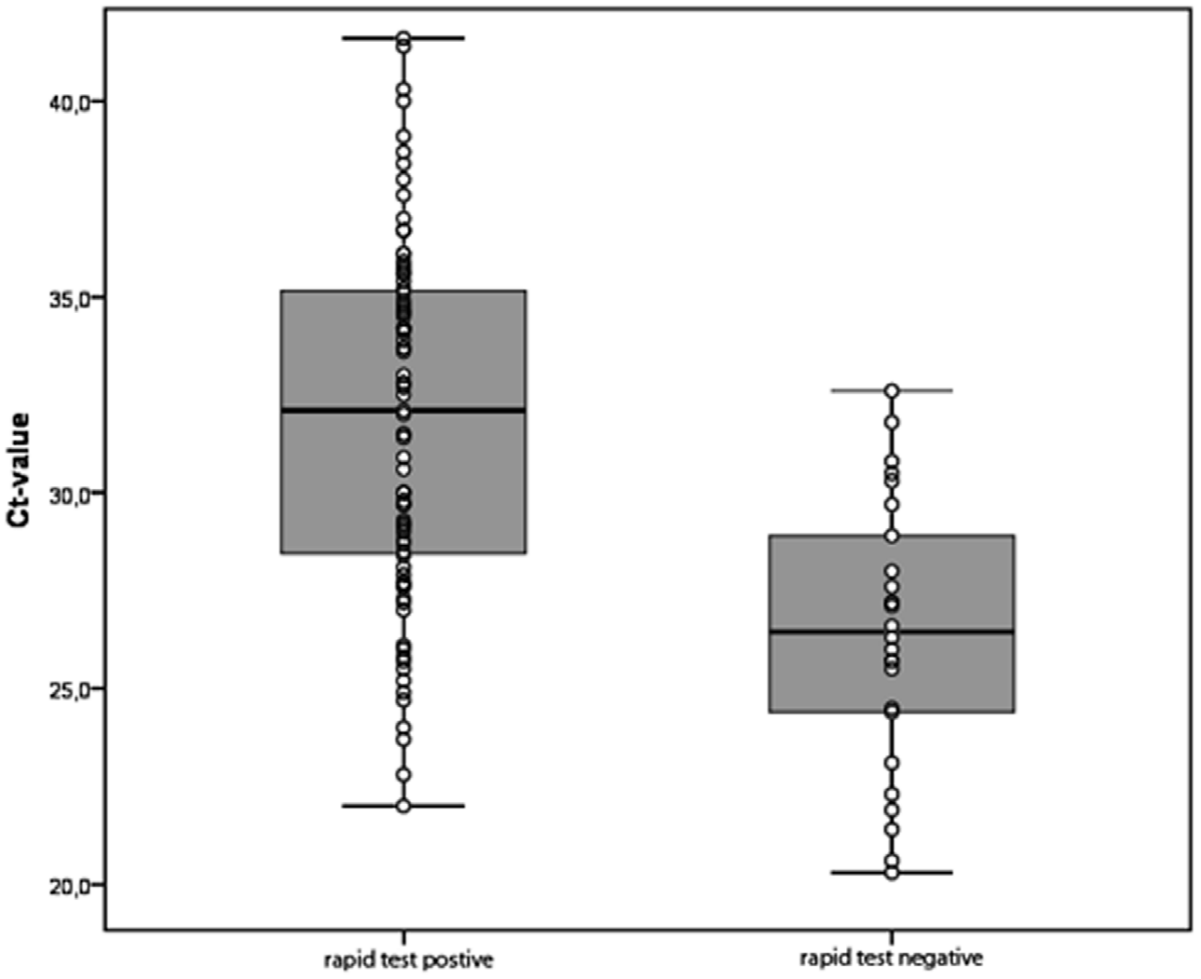
Ct values of respiratory specimens in 119 patients with RT-PCR-confirmed pandemic influenza A H1N1 (2009) virus infections. Ct values are compared between patients who had positive (Ct median 28.44) and negative (Ct median 31.9) results in the QuickVue Influenza A+B rapid test (p<0.001). The box shows the median and interquartile range (box length). The whiskers represent minimum/maximum values. Individual values are presented as circles.

Ct values (median 30.5, minimum 20.3, maximum 41.6) were available for all 119 specimens in which pandemic H1N1 (2009) virus was detected by the RT-PCR. Among these, the median Ct value was 31.9 for 88 specimens with a negative QuickVue test and 28.44 for 31 specimens with a positive test (p<0.001) (Fig. 1). Thus, samples with higher viral loads were more likely to test positive by the rapid test.

**Figure 2:**
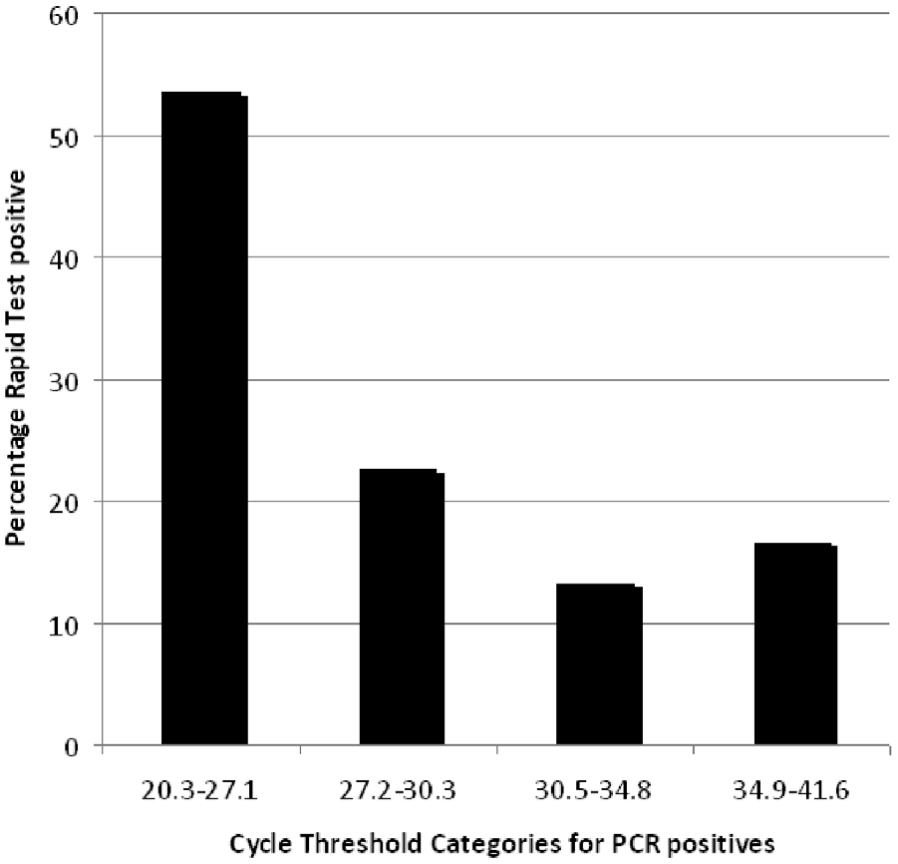
The numbers of specimens with a positive RIDT result were determined within four intervals of cycle threshold (Ct) values: 20.3–27.1, 27.2–30.3, 30.5–34.8, 34.9–41.6 (quartiles of Ct values).

The percentage of rapid test positives for cycle threshold categories is presented in figure 2.

## Discussion

This study was designed to investigate the diagnostic accuracy of the QuickVue Influenza A+B rapid test in adults during the 2009 H1N1 pandemic in Austria. Performance of the rapid test was moderately good, with a low sensitivity (26%) but a high specificity (98%) in comparison with a real time RT-PCR assay.

Previous studies on the QuickVue test for pandemic influenza A H1N1 (2009) have found comparable specificities but higher sensitivities [2], [4], [7], [8], [9], [10], [11], [12], [14]. In a study on comparative epidemiology of pandemic and seasonal influenza A in households performed in Hong Kong a sensitivity of even 80% was reported [15]. The differences in performance of RIDTs in several studies might be due to differences in study design, sample size, and technical factors such as inappropriate specimen collection, transport and storage, and differences between individual patients [9]. The sensitivity found in the present study appears particularly low, possibly due to the smaller sample sizes in earlier studies or, more likely, to the different composition of age categories within our study population. In the present investigation most of the patients were young adults, the age group most severely affected during the 2009 H1N1 pandemic. In addition, no children were enrolled in our study. This is an important point as specimens taken from children tend to have higher influenza viral loads than those taken from adults, which results in better overall sensitivity of RIDTs in specimens from children and makes comparison with studies in patients of different age groups difficult [4].

The high specificity found in our study population is in line with most of the previous studies. In the present study, separate samples were used for the QuickVue test and the RT-PCR, as the swabs provided in the QuickVue kit are unusable for other diagnostic purposes once the swab has been inserted into the kit's testing solution. This could have led to some discordant results and may serve as a possible explanation for the observed test specificity of <100%. However, the recommended sampling procedures were followed for each test, permitting assessment of the diagnostic accuracy of the QuickVue test and the RT-PCR in real-world conditions [7].

The RT-PCR assay used in this study was specific for pandemic influenza A H1N1 (2009) virus and did not include other influenza A virus subtypes such as seasonal influenza A H1N1 or H3N2. To verify the false positive QuickVue results in the two patients with a positive QuickVue result for influenza A but a negative RT-PCR for pandemic influenza A H1N1 (2009), the samples of these two patients were also investigated for other influenza A viruses by PCR analysis. In both samples no infection with seasonal influenza A virus was found, which is in line with national surveillance data demonstrating that the 2009–2010 influenza pandemic in Austria was driven exclusively by pandemic 2009 (H1N1) virus [16].

Despite the low sensitivity found in our study population, specificity was high, resulting in an overall high and positive likelihood ratio. This is important because likelihood ratios are good summary metrics that provide sensible estimates of test properties. In addition, likelihood ratios have the advantage of immediate quantitative clinical utility through direct application of Bayes' theorem [17]. As shown in our study population, the probability of pandemic influenza A H1N1 (2009) virus infection would be high in a patient with a positive QuickVue test, indicating that a positive result with this test does not necessarily need to be confirmed by RT-PCR during influenza outbreaks and suffices to determine the appropriate course of treatment or other action.

In contrast, a negative likelihood ratio of 0.76 as a consequence of the low sensitivity barely alters the probability of an infection in patients with negative QuickVue results. Applying a test with a poor negative likelihood ratio in a high prevalence population underlines the limitations of using RIDTs alone for management of possible pandemic H1N1 virus infection. Reliance on falsely negative test results could delay the diagnosis of H1N1 infection, resulting in inappropriate exposure of susceptible persons to infected patients and the withholding of appropriate therapy. Thus, our data support current opinion that negative RIDT results do not rule out influenza infection and, if relevant for patient management or public health measures such as isolation, negative RIDT results should be confirmed by PCR.

Several reports have found that the sensitivity of an RIDT declines with decreasing viral load in the specimen [2], [4], [6], [18], [19]. Accordingly, we found a higher proportion of positive QuickVue results in patients with higher viral titres (as determined by low Ct values).

Our findings demonstrate that although the QuickVue test is capable of identifying novel influenza H1N1 in respiratory specimens >70% of infections will be missed, particularly in specimens with low viral loads.

The limitations of our study should be noted. The study population in this analysis comprised military personnel and mainly young adults, and the results should not be extrapolated to other age groups. Further, although only patients with influenza-like illness were included, no information was obtained on specific symptoms, severity of symptoms or time elapsed between symptom onset and presentation at the hospital. Thus, differences in performance of the QuickVue test regarding the day of presentation and individual clinical presentation could not be determined.

In conclusion, our data suggest that a positive QuickVue test provides considerable information for the diagnosis of pandemic influenza A 2009 (H1N1) virus infection in young adults, but that negative QuickVue results should, if relevant for patient management or public health measures, be verified with PCR assay.
